# The Impact of Clotting Disorders on Gastrointestinal Health: A Case Report of Bowel Ischemia Due to Thrombus Formation in Antiphospholipid Syndrome and Factor V Leiden

**DOI:** 10.7759/cureus.81556

**Published:** 2025-04-01

**Authors:** Rawan Honeini, Razan Honeini, Mahmoud Abughazal, Noor Ali, Mohammad Hulo

**Affiliations:** 1 Emergency Medicine, United Lincolnshire Hospitals Trust, Boston, GBR; 2 Medicine, Lebanese American University School of Medicine, Beirut, LBN; 3 Accident and Emergency, United Lincolnshire Hospitals Trust, Boston, GBR; 4 Acute Medicine, United Lincolnshire Hospitals Trust, Boston, GBR

**Keywords:** anticoagulant, antiphospholipid antibody, factor v leiden, mesenteric ischemia, venous thrombosis

## Abstract

Antiphospholipid syndrome and Factor V Leiden are well-recognized hypercoagulable disorders associated with an increased risk of thrombosis in major vascular structures. One of the most severe complications is thrombosis of the superior mesenteric vein and its branches, which can lead to impaired blood flow and bowel ischemia. This report presents the case of a 49-year-old woman with underlying hypercoagulable conditions who initially presented with nonspecific gastrointestinal symptoms and unremarkable laboratory findings, ultimately diagnosed with mesenteric venous thrombosis. The case highlights the importance of maintaining a high index of clinical suspicion, facilitating early diagnosis, and ensuring timely intervention to prevent life-threatening complications.

## Introduction

Antiphospholipid syndrome (APS) and Factor V Leiden (FVL) are two prevalent hypercoagulable conditions associated with an increased risk of venous thrombosis. APS is a complex autoimmune disorder characterized by a persistent prothrombotic state, predisposing individuals to recurrent thrombotic events affecting multiple organ systems [1]. It commonly presents with deep vein thrombosis, pulmonary embolism, and arterial thrombosis [2]. In contrast, FVL is an inherited thrombophilia caused by a mutation in Factor V, rendering it resistant to inactivation by activated protein C, a crucial anticoagulant involved in clot regulation. This resistance contributes to an elevated risk of systemic thromboembolism, where FVL was found in about 5-10% of patients presenting with mesenteric venous thrombosis [3]. Here, we present a rare yet clinically significant case of superior mesenteric vein thrombosis (MVT) and its branches in a patient with APS and FVL [4]. Although MVT is uncommon, the coexistence of APS and FVL significantly increases the risk of developing MVT. This report highlights the intricate relationship between clotting disorders and gastrointestinal complications, emphasizing their atypical presentation and the critical need for early recognition and timely intervention.

## Case presentation

A 49-year-old woman with a known history of APS and FVL mutation presented to the emergency department with severe, intermittent cramping abdominal pain, predominantly in the epigastric region. Notably, she had experienced a similar episode one week prior, during which she was diagnosed with gastritis and discharged with a proton pump inhibitor. Despite adherence to the prescribed treatment, her symptoms progressively worsened, with pain intensity reaching 9/10, accompanied by recurrent vomiting and an inability to tolerate oral intake, including fluids. She reported no changes in bowel habits. On examination, her blood pressure was slightly elevated at 143/78 mmHg, and her heart rate was 101 beats per minute. She appeared anxious and in mild distress, unable to sit comfortably. Laboratory tests compared to her previous presentation are shown in Table 1. The results showed an elevated C-reactive protein (CRP) level of 74 mg/L, which was twice the level recorded during the previous presentation, with a similar increase observed in the lactate level. While the white blood cell count, liver function tests, coagulation profile, serum electrolytes, prothrombin time (PT), and international normalized ratio (INR) were within normal limits. However, normal coagulation studies do not rule out the possibility of a thrombotic event, particularly in a patient with existing risk factors. Despite the absence of abnormal coagulation markers, the strong clinical suspicion of MVT persisted, leading to the decision to proceed with imaging.

**Table 1:** Lab parameters Hb: hemoglobin; WBC: white blood cell count; Cr: creatinine; Na: sodium; K: potassium; CRP: C-reactive protein; GFR: glomerular filtration rate; ALT: alanine transaminase

Lab Parameters	Value	Value (Previous Presentation)	Normal Range
WBC	10.6	7.7	4.5-11 x109/L
Platelets	393	271	150-400 x103/µL
Urea	5.5	3.4	2.1 to 8.5 mmol/L
Cr	77	77	59-104 μmol/L
Na	132	138	136 to 145 mmol/L
Hb	15.3	15	14 to 18 g/dL
K	4.1	4.2	3.6-5.2 mmol/L
CRP	74	34	below 5 mg/L
GFR	78	78	90-100 ml/min
Bilirubin	8	8	0-21 μmol/L
ALT	19	16	0-33 U/L
Alkaline Phosphatase	95	100	30-130 U/L
Lactate	1.5	0.7	0.5-2.2 mmol/L

**Figure 1:**
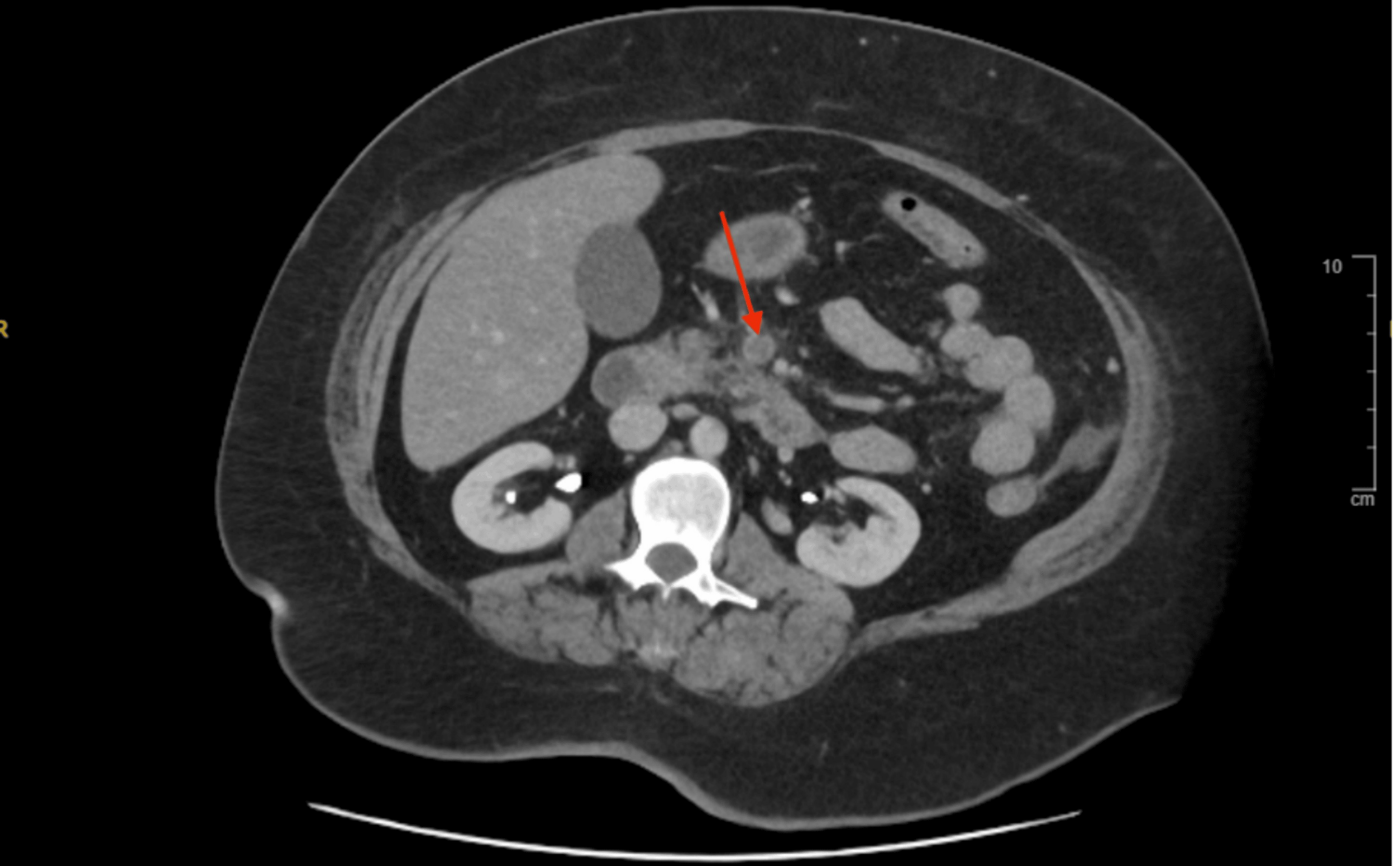
Axial contrast-enhanced CT scan showing acute superior mesenteric vein thrombosis (red arrow)

**Figure 2:**
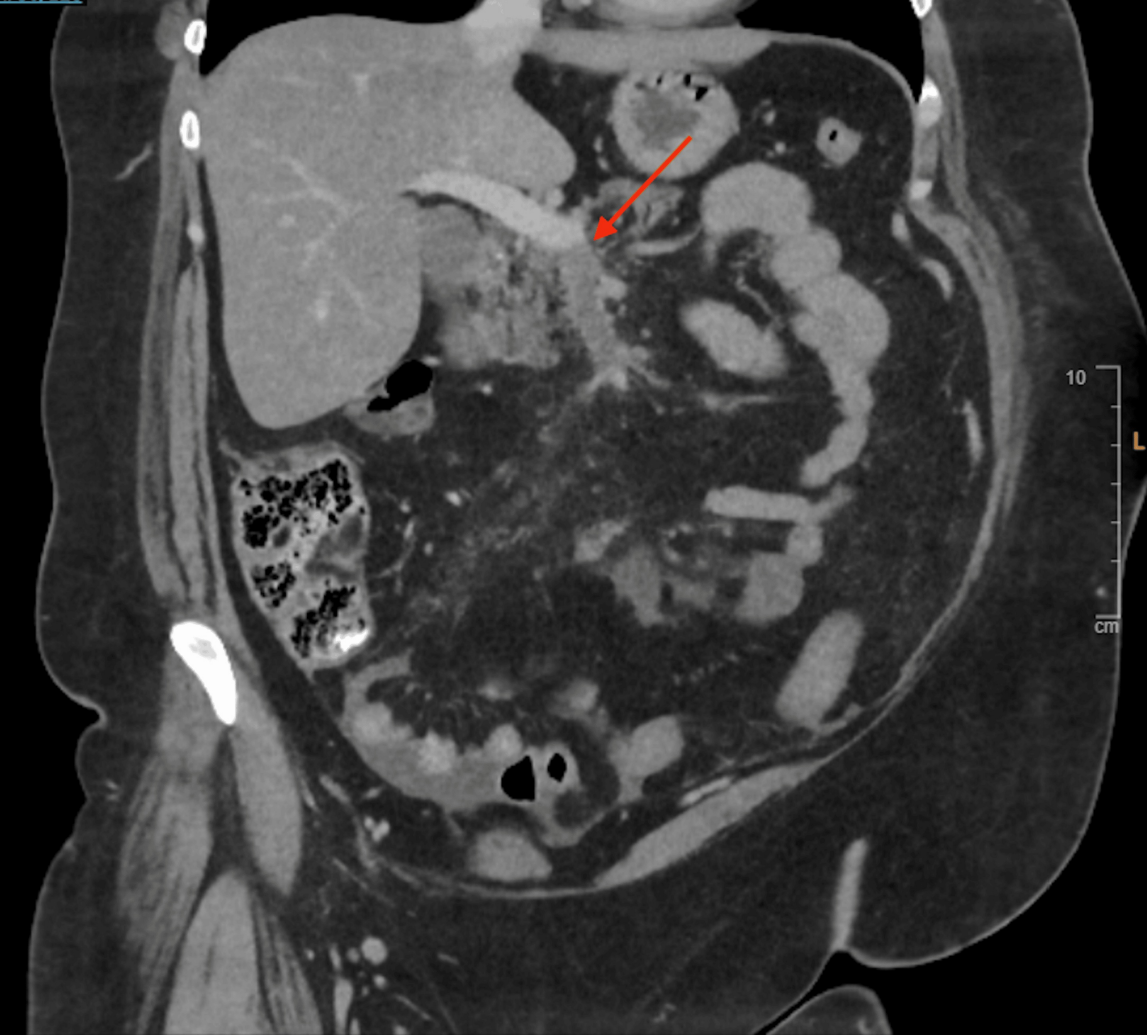
Coronal contrast-enhanced CT scan showing acute superior mesenteric vein thrombosis (red arrow).

Despite receiving intravenous omeprazole, paracetamol, ondansetron, and morphine, her symptoms did not improve. She exhibited severe diffuse abdominal pain disproportionate to physical findings, with a soft but mildly distended abdomen, diffuse tenderness, and normal bowel sounds, without guarding or rebound tenderness. Given her persistent symptoms and underlying hypercoagulable conditions, mesenteric ischemia was suspected. A contrast-enhanced CT scan of the abdomen confirmed an acute thrombus in the superior mesenteric vein, causing complete luminal occlusion. Additionally, stratified wall thickening with peri-enteric fat stranding in segments of the mid-jejunal loops suggested venous bowel ischemia (Figure 1 and Figure 2).

She was admitted to the surgical ward and managed conservatively with antibiotics (ciprofloxacin) and anticoagulation, as recommended by the hematology team. A continuous intravenous infusion of heparin sodium was administered for 10 days, followed by life-long oral apixaban (5 mg twice daily) to prevent further thrombotic events. Transitioning from intravenous (IV) anticoagulation to oral anticoagulants like apixaban is typically done when the patient is clinically stable and has been on therapeutic IV anticoagulation (e.g., heparin or low-molecular-weight heparin) for at least five days or until therapeutic levels were achieved, as indicated by monitoring aPTT or anti-Xa levels. The patient should have no active bleeding, no significant risk of bleeding, and stable renal and hepatic function. Additionally, the patient must not have any contraindications to apixaban (e.g., severe renal impairment, active gastrointestinal bleeding) and should be capable of adhering to oral therapy [5]. Throughout her 10-day hospital stay, the patient was on a clear liquid diet, which was gradually advanced to a regular diet as tolerated. Her condition remained stable, her aPTT was monitored (Table 2), and her CRP and lactate progressively improved (Table 3). By discharge, she was medically optimized, with complete resolution of her presenting symptoms. She was discharged with follow-up plan appointments with hematology in four weeks and general surgery in six to eight weeks.

**Table 2:** Patient's aPTT levels during hospital admission aPTT: activated partial thromboplastin time

Day	Day of Admission	2 Days After Admission	10 Days After Admission	Normal Range
aPTT	30.3	188.3	68.1	26.0 – 35.0 seconds

**Table 3:** Lab parameters on the day of discharge Hb: hemoglobin; WBC: white blood cell count; Cr: creatinine; Na: sodium; K: potassium; CRP: C-reactive protein; GFR: glomerular filtration rate; ALT: alanine transaminase

Lab Parameters	Value (On Day of Discharge)	Normal Range
WBC	8.3	4.5-11 x109/L
Platelets	389	150-400 x103/µL
Urea	5.5	2.1 to 8.5 mmol/L
Cr	77	59-104 μmol/L
Na	137	136 to 145 mmol/L
Hb	12.7	14 to 18 g/dL
K	4.3	3.6-5.2 mmol/L
CRP	2.9	below 5 mg/L
GFR	80	90-100 ml/min
Bilirubin	7	0-21 μmol/L
ALT	20	0-33 U/L
Alkaline Phosphatase	93	30-130 U/L
Lactate	0.6	0.5-2.2 mmol/L

## Discussion

MVT is an uncommon condition, representing 1 in 5,000 to 15,000 hospital admissions, 1 in 1,000 emergency department visits, and 6% to 9% of all acute mesenteric ischemia cases [6-8]. However, its mortality rate can reach 58%, primarily due to nonspecific symptoms and delayed diagnosis [9]. The superior mesenteric vein is the most frequently affected vessel [4]. Historically, MVT was often diagnosed intraoperatively or postmortem due to its vague clinical presentation and the absence of definitive early symptoms [8]. However, advancements in diagnostic imaging have significantly improved early detection rates. Furthermore, the proportion of cases classified as idiopathic has declined with routine screening for hypercoagulable conditions, allowing for better risk stratification and earlier intervention [8].

This case highlights the intricate interplay between thrombotic disorders and atypical clinical presentations, as demonstrated by the patient’s initial misdiagnosis as gastritis. MVT often presents with recurrent abdominal pain, vomiting, and food intolerance, which can mimic primary gastrointestinal pathologies. However, pain that appears disproportionate to physical examination findings, particularly in patients with known hematologic disorders, should raise clinical suspicion for an underlying thromboembolic event.

Risk factors for mesenteric venous thrombosis include coagulation disorders, cancer, recent surgical procedures, abdominal inflammation, such as inflammatory bowel disease, pancreatitis, and diverticulitis, and local venous congestion like cirrhosis [10]. Hypercoagulable states, whether congenital or acquired, are major predisposing factors for MVT. In APS, recurrent thrombotic events are common despite anticoagulation therapy, with an estimated annual recurrence rate of 5-12% [11]. A comprehensive meta-analysis found that individuals with FVL have a 1.4 to 2.4 times higher risk of VTE recurrence than those without the mutation [12]. Our patient exhibited two such risk factors: APS and FVL mutation. In APS, antiphospholipid (aPL) antibodies contribute to a hypercoagulable state through multiple mechanisms, including enhanced platelet aggregation, increased pro-inflammatory cytokine production (e.g., interleukin-6 and tumor necrosis factor-alpha), and upregulation of endothelial glycoprotein IIb/IIIa expression [13]. Gastrointestinal involvement in APS can manifest as MVT, with potential progression to bowel ischemia and infarction. Similarly, FVL mutation leads to resistance to activated protein C, disrupting the balance between pro- and anticoagulation mechanisms and increasing the risk of thrombus formation [14]. While FVL is more commonly associated with systemic thromboembolism, it can also contribute to localized thrombosis in critical vascular territories such as the superior mesenteric vein. Notably, studies estimate that approximately 6% of patients with FVL develop MVT, with all cases involving the superior mesenteric vein [14]. In this patient, the coexistence of APS and FVL significantly heightened the risk of thrombotic complications.

Unlike acute mesenteric ischemia caused by arterial occlusion, which typically presents with sudden and severe symptoms, MVT often follows a more insidious course [4]. Patients generally experience symptoms for an average of 5 to 14 days before seeking medical attention, with over 75% reporting at least 2 days of abdominal pain [4]. The hallmark feature of mesenteric ischemia is abdominal pain that appears out of proportion to physical examination findings. However, the clinical presentation of MVT varies widely depending on the affected segment, the extent of thrombosis, and the degree of ischemia. Our patient exhibited a subacute thrombotic event that escalated rapidly. The persistence of severe cramping abdominal pain and vomiting, despite prior treatment for gastritis, underscores the importance of a comprehensive diagnostic evaluation in patients with known hypercoagulable disorders presenting with gastrointestinal symptoms.

Early diagnosis and prompt management are crucial in preventing severe complications such as bowel necrosis and the need for surgical intervention. Anticoagulation therapy remains the cornerstone of treatment, with studies reporting a recanalization rate of up to 38% following timely initiation of therapy [15]. Management of MVT includes bowel rest, intravenous fluid resuscitation, analgesia, and anticoagulation to prevent thrombus propagation and facilitate recanalization [16,17]. Low-molecular-weight heparin is generally preferred over unfractionated heparin for initial anticoagulation due to its superior safety profile and efficacy [18]. Surgical intervention is reserved for patients with bowel ischemia or complications such as bowel perforation [19]. Beyond acute management, long-term anticoagulation is essential to mitigate the risk of recurrent thrombosis. In this case, the patient was initially managed with intravenous heparin, followed by long-term anticoagulation with apixaban to address both the acute thrombotic event and the underlying hypercoagulable state, ensuring comprehensive thrombotic risk management.

## Conclusions

In summary, this case highlights the importance of keeping a high index of suspicion for mesenteric ischemia in patients with clotting disorders, particularly when vague gastrointestinal symptoms are reported. Although MVT is rare, its subsequent morbidity and mortality rates warrant early detection and timely intervention.
